# Treatment of hyponatremia in children with acute bacterial meningitis

**DOI:** 10.3389/fneur.2022.911784

**Published:** 2022-08-10

**Authors:** Feixia Zheng, Xiaoyan Ye, Yuanyuan Chen, Hongying Wang, Shiyu Fang, Xulai Shi, Zhongdong Lin, Zhenlang Lin

**Affiliations:** ^1^Department of Pediatrics, The Second Affiliated Hospital and Yuying Children's Hospital of Wenzhou Medical University, Wenzhou, Zhejiang, China; ^2^Department of Pediatrics, Aksu First People's Hospital, Xinjiang, China

**Keywords:** hypernatremia, pediatric, prognosis, treatment, dysnatremia

## Abstract

**Purpose:**

Few studies have evaluated hyponatremia management in children with bacterial meningitis (BM). Thus, we aimed to describe variations in clinical practice, the effectiveness of sodium management, and adverse outcomes in children with BM and hyponatremia.

**Methods:**

This retrospective cross-sectional study conducted at a tertiary institution analyzed participants' demographic, clinical, and sodium-altering treatment data. The sodium trigger for treatment was defined as pretreatment sodium level, with response and overcorrection defined as increments of ≥5 and >10 mmol/L after 24 h, respectively.

**Results:**

This study enrolled 364 children with BM (age: <16 years; 215 boys). Hyponatremia occurred in 62.1% of patients, among whom 25.7% received sodium-altering therapies; 91.4% of those individuals had moderate/severe hyponatremia. Monotherapy was the most common initial hyponatremia treatment. After 24 h of treatment initiation, 82.4% of the patients responded. Logistic regression analyses revealed that ΔNa^24^ <5 mmol/L [odds ratio (OR) 15.52, 95% CI 1.71–141.06, *p* = 0.015] and minimum Glasgow Coma Scale (GCS) score ≤ 8 (OR 11.09, 95% CI 1.16–105.73, *p* = 0.036) predicted dysnatremia at 48 h after treatment initiation. Although rare, persistent moderate/severe hyponatremia or hypernatremia at 48 h after treatment initiation was associated with a high mortality rate (57.1%).

**Conclusion:**

This study found that most cases of hyponatremia responded well to various treatments. It is important to identify and institute appropriate treatment early for moderate or severe hyponatremia or hypernatremia in children with BM. This study was limited by its non-randomized nature.

## Introduction

Serum sodium (Na^+^) concentration is an essential determinant of several nervous system functions, and rapid increases or decreases in the sodium level can cause permanent, severe, and occasional lethal brain injury; furthermore, improper adjustments of sodium levels can be detrimental (1). Generally, inflammation affects plasma sodium, and several acute inflammatory disorders lead to hyponatremia, especially in children (2). Dysnatremia, particularly hyponatremias, is the most common electrolyte disorder in patients with bacterial meningitis (BM). The reported incidence rates of hyponatremia (Na^+^ <135 mmol/L) at hospital admission for BM range from 30.3 to 66.4% (3–5), whereas hypernatremia (Na^+^ ≥146 mmol/L) is ~2% in adults with BM (6).

The pathophysiology of hyponatremia is complex. Dehydration, iatrogenic factors, syndrome of inappropriate secretion of antidiuretic hormone, and cerebral salt wasting syndrome result in remarkable volume loss that may contribute to the etiology of hyponatremia in neurological diseases (7, 8) Hypertonic fluid gain for the treatment of hyponatremia, diabetes insipidus, and osmotic diarrhea can cause hypernatremia in BM (9). Severe hyponatremia (Na^+^ <125 mmol/L) and hypernatremia are markers of acute disease severity, morbidity, and unfavorable outcomes in meningitis. Careful management of fluid and electrolyte balance is important in the treatment of meningitis (4, 6, 7, 10, 11).

Treatment of hyponatremia varies according to the type of neurological disease (12). For instance, fluid restriction should be avoided in severe stroke and traumatic brain injury, as euvolemia is recommended for maintaining adequate cerebral perfusion pressure in these patients (13). However, fluid restriction is widely advocated in the initial treatment of pediatric meningitis based on reports that attribute hyponatremia to increased concentrations of circulating antidiuretic hormone (7, 14). To a large extent, the signs and symptoms of dysnatremia overlap with those of BM and its complications, such as subdural fluid accumulation; therefore, the assessment of symptom severity in dysnatremia is often inaccurate (11). Different strategies may be required to manage sodium levels of patients with BM and hyponatremias than for the general population with ill health or other neurological diseases.

Few studies have evaluated the management protocols of hyponatremia in children with BM (15). This study aimed to describe the current practices, the effectiveness of sodium management, and adverse clinical outcomes in the treatment of hyponatremia in pediatric BM.

## Methods

### Study population

A total of 364 patients (210 boys and 154 girls) aged 1 day to 16 years, admitted to the Department of Pediatrics between 1 December 2015 and 1 July 2021 for BM, were included in this retrospective study. BM diagnosis was based on the detection of bacteria in the cerebrospinal fluid (CSF) by culture analysis or Gram staining. Furthermore, CSF pleocytosis with predominant neutrophils, hypoglycorrhachia, and increased protein levels were also considered to be indicators of BM (4, 16). Patients with conditions, such as primary renal, hepatic, or cardiac failure; pulmonary disease; or other neurological diseases that could result in dysnatremia in the most recent 6 months, were excluded from the present study.

### Ethical statement

The study was approved by the Research Ethics Board at the Second Affiliated Hospital of Wenzhou Medical University. The requirement for informed consent was waived as the data were anonymous and the study was retrospective (2022-K-25-01).

### Definition of dysnatremia and normonatremia

Hyponatremia, normonatremia, and hypernatremia were defined as serum Na^+^ levels <135, 135–145, and >145 mmol/L, respectively (17–19). The values were assigned based on the minimum and maximum levels of serum sodium at the time of admission and during hospitalization. The severity of hyponatremia was further graded as mild, 130–135 mmol/L; moderate, 125–129 mmol/L; and severe, <125 mmol/L (4, 9, 17).

### Investigations

Total/peripheral parenteral nutrition, 10% dextrose in 0.33% normal saline (NS), and 10% dextrose in 0.20% NS were used as maintenance fluids when necessary (4). Sodium-altering therapies for hyponatremia included intravenous (IV) fluids and oral sodium chloride supplements; IV fluids used to correct hyponatremia included hypertonic (including 3% NaCl), isotonic [including normal saline (NS), D5W NS, and 1.4% sodium bicarbonate], and hypotonic saline (including sodium chloride supplementation through total/peripheral parenteral nutrition and D10W-one-half NS), and fluid restriction. Fluid restriction or fluid administration was used as sodium-altering therapies and was determined using electronic records. Combination therapy was defined as the use of two or more sodium-altering therapies (oral sodium chloride supplements, IV hypertonic saline, IV isotonic saline, IV hypotonic saline, or fluid restriction) in one patient. The sodium level prompting the decision to initiate sodium-altering therapy was designated as the “trigger sodium” value.

### Evaluation

Considering time zero (Na^0^) as the time of initiation of sodium-altering therapies after the detection of the trigger sodium value, the sodium correction rate (mmol/L per day) was estimated after 24 h using the following formula:


$$\begin{array}{l} \text{N}{\text{a}}^{\text{24}}=\text{N}{\text{a}}^{\text{a}}+\left[\left(\text{N}{\text{a}}^{\text{b}}-\text{N}{\text{a}}^{\text{a}}\right)\times \left(\text{24}-{\text{T}}_{\text{a}}\right)/\left({\text{T}}_{\text{b}}-{\text{T}}_{\text{a}}\right)\right] \end{array}$$

where Na^a^ and Na^b^ indicate the two most recent Na^+^ measurements before and after 24 h, respectively, and T_a_ and T_b_ are the times at which Na^a^ and Na^b^ were measured, respectively. The Na^+^ correction rate was calculated as follows: ΔNa^24^ = Na^24^ – Na^0^. Sodium response was defined as a sodium correction rate of ≥5 mmol/L per day, which was the primary outcome of the study. Patients were considered not to have responded if their sodium correction rate was <5 mmol/L per day and to have experienced overcorrection if their sodium correction rate was >10 mmol/L at any time during the first 24 h after treatment initiation (20). If patients experienced more than one episode of hyponatremia during the study period, only the most severe episode was considered in the final analysis.

Treated patients who were normonatremic 48 h after the onset of sodium-altering therapy were classified as having corrected hyponatremia; those with mild hyponatremia were classified as having partly corrected hyponatremia, while those with moderate or severe hyponatremia (Na^+^ <130 mmol/L) or hypernatremia as having uncorrected dysnatremia. Disease severity was determined using the maximum Pediatric Cerebral Performance Category (PCPC) and minimum Glasgow Coma Scale (GCS) values. A short-term prognosis was determined using PCPC at discharge. Adverse clinical outcomes were defined as death or a PCPC value of ≥2 at discharge. The secondary outcomes of the study were based on these clinical parameters.

### Statistical analysis

All statistical analyses were performed using SPSS version 25 (IBM Corporation, Armonk, NY, USA). Categorical variables were evaluated using Fisher's exact test or the chi-square test. Non-normally distributed data were evaluated using the Mann–Whitney *U*-test or the Kruskal–Wallis *H*-test. Continuous variables were summarized as medians and interquartile ranges (IQRs). Factors predictive of dysnatremia at 48 h after treatment initiation were assessed *via* univariable binary logistic regression. Statistical significance was set at *p* < 0.05.

## Results

### Demographics

**Figure 1:**
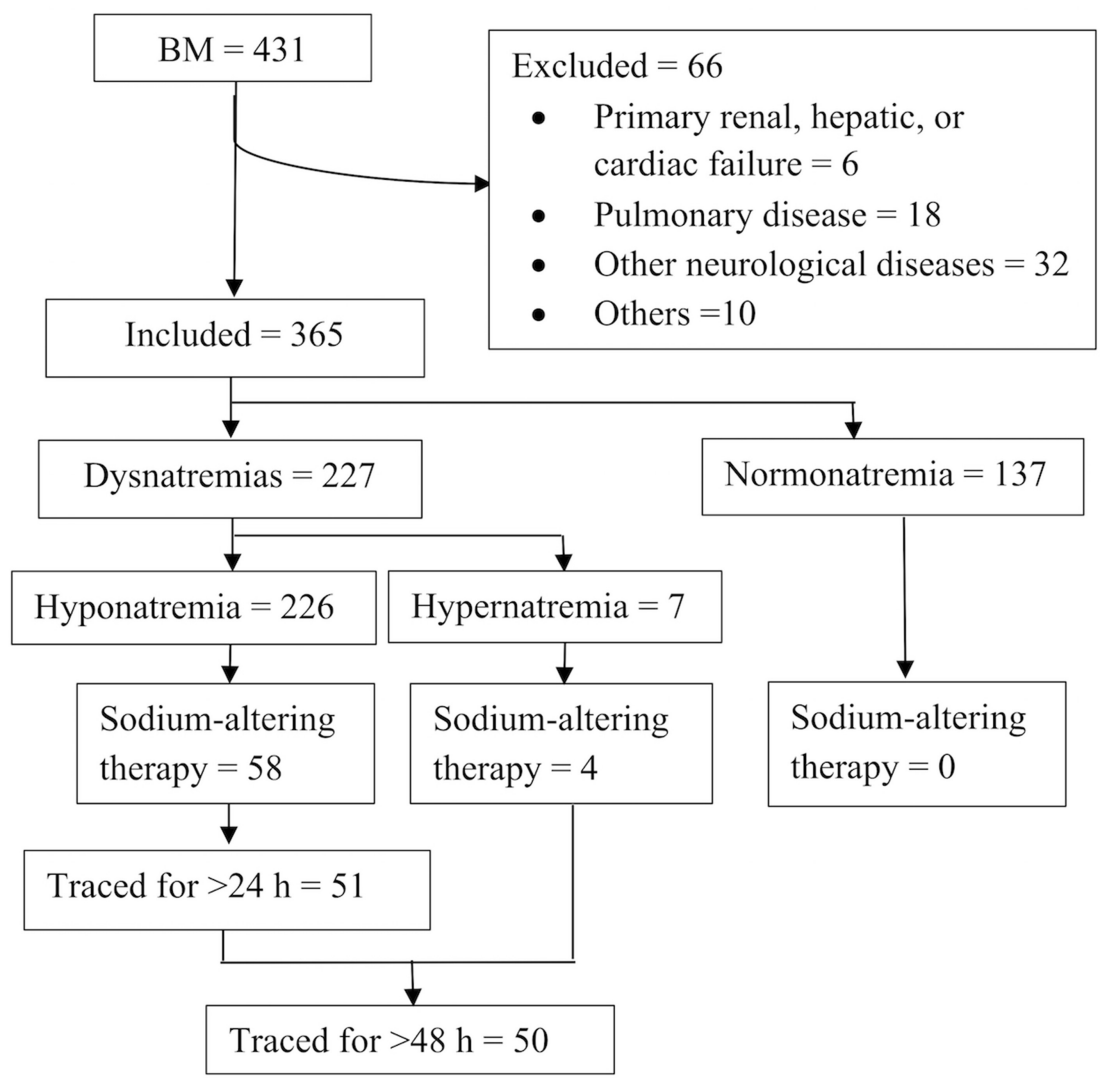
Study flowchart.

The patient selection flowchart is shown in Figure 1. Among 364 patients with BM, 226 (62.1%) had hyponatremia while 7 (1.9%) had hypernatremia. Hyponatremia therapy was initiated in 15.9% (58/364) of the patients. None of the patients with normonatremia received sodium-altering therapies. The characteristics of patients with hyponatremia are summarized in Table 1. A higher proportion of neonates with hyponatremia received treatment in comparison to non-neonates. Patients with severe hyponatremia were also more likely to be treated (mild vs. moderate vs. severe: 2.6 vs. 68.8 vs. 91.3%). Although treated patients had significantly greater disease severity and a higher frequency of seizures than untreated patients, the only indication for treatment was a declining sodium level (58/58, 100%).

**Table 1:** Characteristics of patients with hyponatremia.

**Characteristic**		**Hyponatremia** **treatment**	**No hyponatremia** **treatment**	**Total**	***p*** **-Value**
*n*		58	168	226	
Age	Neonates (≤ 28 days)	30/88 (34.1%)	58	88	
	>28 days	28/138 (20.3%)	110	138	0.021
Sex	Male	37/126 (29.3%)	89	126	
	Female	21/100 (21%)	79	100	0.153
Etiological organism	*E. coli*	7/21 (33.3%)	14	21	
	*S. pneumoniae*	3/10 (30%)	7	10	
	*S. agalactiae*	17/34 (50%)	17	34	
	Others	6/20 (30%)	14	20	0.412
Seizure		25 /58 (43.1%)	25 /168 (14.9%)		<0.001
Severity of hyponatremia	Mild	4/155 (2.6%)	151	155	
	Moderate	33/48 (68.8%)	15	48	
	Severe	21/23 (91.3%)	2	23	<0.001
Severity of disease	Minimum GCS	14 (11, 14)	15 (14, 15)		<0.001
	Maximum PCPC	2 (1, 4)	1 (1, 2)		<0.001
Poor outcome	PCPC on discharge ≥2	9/58 (15.5%)	2/168 (1.2%)		<0.001

S. pneumoniae, Streptococcus pneumoniae; S. agalactiae, Streptococcus agalactiae; E. coli, Escherichia coli; GCS, Glasgow Coma Scale; PCPC, Pediatric Cerebral Performance Category.

### Trigger sodium for hyponatremia therapy

The majority of patients (53/58, 91.4%) who received sodium-altering therapy had a trigger sodium level of <130 mmol/L. The median trigger sodium level was 126.2 mmol/L (IQR: 124.1, 128.5 mmol/L), and it did not differ significantly between neonates and non-neonates [126.0 (IQR: 123.8, 128.7) vs. 126.7 (IQR: 124.9, 128.5) mmol/L, *p* = 0.431]. The trigger sodium level of patients who received combination therapy initially was markedly lower than that of patients who received monotherapy initially [123.6 (IQR: 120.9, 125.7) vs. 127.8 (IQR: 125.0, 128.9) mmol/L, *p* = 0.006].

### Hyponatremia therapy

Sodium-altering therapies administered to patients with hyponatremia are summarized in Table 2. Among the 58 treated patients, 45 (75.9%) received monotherapy initially and 13 (24.1%) received two treatment modalities. A second-line agent was later administered to 10 (17.2%) patients who received monotherapy initially. Hypertonic saline was the most common treatment for hyponatremia, followed by oral sodium chloride, isotonic saline, and hypotonic saline. Fluid restriction was rarely used as a sodium-altering therapy. As the treatment strategy used for neonates differed from those used for non-neonates, we analyzed these groups separately. Oral sodium chloride and hypertonic saline were the most common initial treatments in neonates, whereas isotonic saline was the most common initial treatment in non-neonates. The most common initial combination therapy included oral sodium chloride supplement and IV hypertonic saline (5/30, 16.7%) in neonates and a combination of IV hypertonic and isotonic saline (4/28, 14.3%) in non-neonates. Hypertonic saline was the most common second-line agent used.

**Table 2:** Sodium-altering therapy employed as first or second-line treatment.

	**Initial (** ***n*** **)**		**Secondary (*n*)**	**Anytime (*n*)**
	**Monotherapy**	**Combination therapy**		
**Neonates (*****n*** **=** **30)**	23 (76.7%)	7 (23.3%)	7 (23.3%)	
Oral sodium chloride	14 (46.7%)	6 (20%)	1 (3.3%)	21 (70%)
Hypertonic saline	5 (16.7%)	6 (20%)	5 (16.7%)	16 (53.3%)
Isotonic saline	2 (6.7%)	1 (3.3%)	0	3 (10%)
Hypotonic saline	2 (6.7%)	1 (3.3%)	1 (3.3%)	4 (13.3%)
**Children beyond neonatal age (*****n*** **= 28)**	22 (78.5%)	6 (21.4%)	3 (10.7%)	
Oral sodium chloride	0	0	0	0
Hypertonic saline	2 (7.1%)	4 (14.3%)	2 (7.1%)	8 (28.6%)
Isotonic saline	15 (53.3%)	6 (21.4%)	1 (3.6%)	22 (78.6%)
Hypotonic saline	5 (17.9%)	2 (7.1%)	0	7 (25%)
**Overall (*****n*** **= 58)**	45 (75.9%)	13 (24.1%)	10 (15.5%)	
Oral sodium chloride	14 (24.1%)	6 (10.3%)	1 (1.7%)	21 (36.2 %)
Hypertonic saline	17 (29.3%)	10 (17.2%)	7 (12.1%)	28 (48.3 %)
Isotonic saline	7 (12.1 %)	7 (12.1%)	1 (1.7%)	15 (25.9 %)
Hypotonic saline	7 (12.1%)	3 (5.2%)	1 (1.7%)	11 (19.0%)

### Twenty-four-hour therapeutic efficacy

Among the 58 treated patients, 51 underwent a follow-up serum sodium measurement ~24 h after the initiation of sodium-altering therapies (Table 3). Of these patients, 82.4% (42/51) responded to the sodium-altering therapies within 24 h, with a median sodium level increase (ΔNa^24^) of 7.6 mmol/L (IQR: 5, 10.5 mmol/L). In total, 70.6% (36/51) of the patients responded to the initial sodium-altering therapies. Hyponatremia was overcorrected in a minority of patients (27.5%, 14/51). There were no significant differences in ΔNa^24^, response rate, or overcorrection rates between patients who received monotherapy and combination therapy or between neonates and non-neonates. None of these patients presented new neurological symptoms or signs indicating osmotic demyelination syndrome after sodium-altering therapies. Evaluation of each monotherapy revealed response and overcorrection rates of 100 and 40% for IV hypertonic saline, 91.7 and 8.5% for isotonic saline, 85.7 and 28.6% for oral sodium chloride, and 50 and 25% for hypotonic saline. Hyponatremia worsened in 28.6% (2/7) of the patients after initial hypotonic saline treatment and in 28.6% (2/7) of patients after initial isotonic saline treatment, before second-line treatment was administered.

**Table 3:** Sodium response, overcorrection, and 24-h change in sodium with treatment.

	**Total (*n*)**	**Sodium response**	**Overcorrection**	**ΔNa^24^ (mmol/L)**
**Overall (*****n*** **= 51)**				
Monotherapy	28	24 (85.7%)	6 (21.4%)	7.4 (IQR 4.9, 10)
Combination therapy	23	18 (78.3%)	8 (34.8%)	7.9 (IQR 5.5, 14.1)
*p*-Value		0.714	0.288	0.616
**Age**				
Neonates (≤ 28 days)	25	22 (88%)	7 (28%)	7.6 (IQR 5.7, 10.4)
>28 days	26	20 (76.9%)	7 (26.9%)	7.7 (IQR 4.4, 11.6)
*p*-Value		0.465	0.931	0.932
**Monotherapy (*****n*** **= 28)**				
Oral sodium chloride	7	6 (85.7%)	2 (28.6%)	8.6 (IQR 4.1, 10.3)
Hypertonic saline	5	5 (100%)	2 (40%)	7 (IQR 6.7, 18)
Isotonic saline	12	11 (91.7%)	1 (8.5%)	7.7 (IQR 5.5, 9.1)
Hypotonic saline	4	2 (50%)	1 (25%)	4.75 (IQR 3.2, 9.9)

Δ.a^24^, change in sodium over 24 h.

### Forty-eight-hour treatment outcome

Serum sodium progression was traced for >48 h in 50 treated patients (Table 4). Most patients (26/50, 52%) had corrected hyponatremia [Na^+^: 136.6 (IQR: 136, 137.9) mmol/L]. Nineteen patients (38%) had partly corrected hyponatremia, with mild hyponatremia [Na^+^: 133.5 (IQR: 131.5, 134.2) mmol/L] persisting for 2–10 days with or without sodium-altering therapies. A minority of patients (5/50, 10%, patients 2–6) had uncorrected dysnatremia; who responded well to hyponatremia treatment at 24 h after treatment initiation and experienced both hypernatremia [Na^+^: 146.7 (IQR: 146.2, 153.6) mmol/L] and hyponatremia, whereas two patients (4%) experienced moderate or severe hyponatremia persisting for >2 days (which was occasionally resistant to IV saline). Uncorrected dysnatremia, compared with partly corrected hyponatremia and corrected hyponatremia, was associated with a significantly higher frequency of severe brain injury (minimum GCS score ≤ 8 and maximum PCPC score), respiratory failure required invasive mechanical ventilation, shock, hypernatremia, and death or poor short-term prognosis. Multivariate analyses revealed that ΔNa^24^ <5 mmol/L (OR 15.52, 95% CI 1.71–141.06, *p* = 0.015) and minimum GCS score ≤ 8 (OR 11.09, 95% CI 1.16–105.73, *p* = 0.036) were related to dysnatremia at 48 h after treatment initiation.

**Table 4:** Comparison of characteristics of bacterial meningitis patients with corrected hypernatremia, partly corrected hypernatremia, and uncorrected hyponatremia.

**Patient characteristic**	**Corrected hyponatremia**	**Partly corrected hyponatremia**	**Uncorrected dysnatremia**	***p-*** **Value**
Age (years)	0.08 (0.04, 0.25)	0.07 (0.04, 0.17)	0.07 (0.03, 0.12)	0.769
Female	15/26 (57.4%)	11/19 (57.9%)	4/5 (80%)	0.832
Convulsions (*n*)	11/26 (42.3%)	9/19 (47.4%)	4/5 (80%)	0.301
GCS minimum score ≤ 8	1/26 (3.8%)	1/19 (5.3%)	5/5 (100%)	0.000
PCPC maximum score	2 (1.75, 3)	2 (1, 3)	5 (4.5, 6)	0.001
**Laboratory tests**				
Minimum serum sodium concentration (mmol/L)	126.2 (124, 128.03)	125.5 (123.6, 128.7)	121.5 (112, 129.6)	0.691
Hypernatremia (*n*)	1/26 (3.8%)	0/19 (0%)	5/5 (100%)	0.000
**Indexes of CSF**				
White cell count (/10^9^L)	894.5 (182.5, 3,285)	325 (80, 2,400)	260 (83, 8,069.5)	0.476
Protein >3 g/L	10/25 (40%)	9/19 (47.4%)	4/5 (80%)	0.269
**Clinical complications**				
Shock (*n*)	3/26 (11.5%)	3/19 (15.8%)	3/5 (60%)	0.045
MODS (*n*)	2/26 (7.7%)	1/19 (5.3%)	2/5 (40%)	0.086
Mechanical ventilation (*n*)	2/26 (7.7%)	1/19 (5.3%)	3/5 (60%)	0.017
Hydrocephalus (*n*)	1/25 (4%)	1/19 (5.3%)	0/5 (0%)	1.000
Subdural fluid accumulation (*n*)	2/25 (8%)	6/19 (31.6%)	2/5 (40%)	0.047
Subdural empyema (*n*)	1/25 (4%)	0/19 (0%)	1/5 (20%)	0.196
Brain parenchymal abnormalities (*n*)	4/25 (16%)	5/19 (26.3%)	3/5 (60%)	0.100
**Treatment**				
Combination therapy (*n*)	5/26 (19.2%)	6/19 (31.6%)	2/5 (40%)	0.520
Sodium response (*n*)	25/26 (96.2%)	11/19 (57.9%)	5/5 (100%)	0.003
Overcorrection (*n*)	9/26 (34.6%)	2/19 (10.5%)	3/5 (60%)	0.038
**Outcomes**				
Poor outcome (PCPC ≥2)	0/26 (0%)	2/19 (10.5%)	5/5 (100%)	0.000

Six patients with hyponatremia experienced hypernatremia before or after an episode of hyponatremia, and in one patient, only hypernatremia was detected as the patient withdrew from treatment due to severe disease conditions 1 day after admission. Hypernatremia was due to central diabetes insipidus in three patients, all of whom received pitressin or desmopressin after disease confirmation. No hypernatremia caused specifically by hypertonic fluid gain was observed. Hypernatremia persisted for 1–60 days.

## Discussion

In the present study, hyponatremia therapy was employed in 15.9% of the children with BM, most of whom (91.4%) had a trigger sodium level of <130 mmol/L. The treatment choices for hyponatremia varied, with 82.4% of the patients showing a positive response at 24 h. Monotherapy was the most common initial hyponatremia treatment employed. Hypertonic saline was the most common second-line agent administered. A negative sodium response at 24 h after treatment initiation and minimum GCS score of ≤ 8 were related to dysnatremia at 48 h after treatment. Only a minority of the patients experienced persistent moderate or severe hyponatremia or hypernatremia at 48 h after treatment initiation, and most of these experienced adverse clinical outcomes.

A higher median trigger sodium level was observed in patients with neurological injury in a previous study than that in pediatric patients with BM in the present study (126.2 vs. 133 mmol/L) (12). This may have been due to the widely advocated recommendation of fluid restriction during the initial treatment of pediatric meningitis; however, this approach has been challenged. The cause of hyponatremia is often unclear (21). This is also true in the present study in which the cause of hyponatremia was diagnosed in only a few chronic moderate or severe cases. The role of dehydration or inappropriate increment of vasopressin levels as the key contributors to the pathogenesis of hyponatremia in meningitis is debatable (7). In patients with meningitis, increased antidiuretic hormone concentrations may be due to hypovolemia, which can only be corrected with the administration of sufficient fluid and sodium (22). Guidelines on the preference of maintenance fluids over fluid restriction in the treatment of acute BM have not been set up, due to lack of sufficient data (7). Hypotonic maintenance fluids were used in these studies since they have been widely advocated to be appropriate for infants and children. In 2018, the American Academy of Pediatrics recommended that non-neonates requiring maintenance IV fluids should receive isotonic solutions to decrease the risk of hyponatremia (23). However, further research is needed to investigate if the change in the recommended maintenance of IV fluids would provide sufficient evidence on appropriate fluid therapy in the management of pediatric BM.

The choice of treatment for hyponatremia varied between the current study and a previously reported study on adults with neurological injuries (12), although the initial monotherapy was common in both the studies (12). Combination therapy did not demonstrate a more robust response when compared to monotherapy in the current study. This may be because combination therapy was more often used in the treatment of severe hyponatremia. Hypertonic saline has been reported to provide a more consistent sodium level increase than conventional strategies in adults with neurological injury and among the general population with ill-health (12, 24). In the present study, hypertonic saline was the most common second-line agent administered and was the only IV fluid that did not show worsening of hyponatremia after monotherapy. However, it is unclear if it had a more robust response than other strategies in children with BM.

In clinical practice, suboptimal correction of severe hyponatremia is common, with reported incidences of 21 and 21–27.9% for under-corrected and overcorrected hyponatremia, respectively (20, 24). In the present study, the rate of patient response to sodium-altering therapies was 82.4%, relative to the 60% reported in adults with neurological injury (12). Overcorrection was also frequent in the present study but was not associated with the clinical features of osmotic demyelination syndrome [which can be caused by rapid correction of chronic hyponatremia (1, 25–27)], possibly because most corrections in our patients were occurred during acute hyponatremia. However, neuroimaging was not routinely performed in these patients to definitively exclude osmotic demyelination syndrome, which is a limitation of our study.

Dysnatremia correction is independently associated with patient survival. Previous retrospective studies have revealed that persistent hyponatremia on day 3 and persistent hypernatremia (>24–72 h) persistent were independently associated with higher mortality in hospitalized patients (28, 29). In the present study, most dysnatremia cases were corrected within 48 h. A negative sodium response at 24 h after treatment initiation and a minimum GCS score ≤ 8 were related to dysnatremia at 48 h after treatment initiation. Persistent moderate or severe hyponatremia and hypernatremia at 48 h were rarely occurred but were associated with increased disease severity, higher mortality, and poor survivor prognosis in patients with BM.

The miscellaneous clinical data are the strengths of our study. However, this study has several limitations. Treatment decisions were made at the discretion of the pediatricians in the pediatric neurology department, neonatology department, pediatric emergency department, and intensive care unit. Moreover, there was no randomization, and total fluid or sodium intake measurements were unavailable. Prospective studies with larger numbers of patients may indicate possible differences in the sodium-altering effects and patient outcomes for various treatments and may confirm the relationship between hyponatremia correction and adverse clinical outcomes. In addition, we did not study neurodevelopmental outcomes, due to data unavailability. Hence, the effectiveness of individual treatment modalities was not evaluated in terms of divergence in outcomes.

Thus, to conclude, we found that hyponatremia therapy was uncommon, and methods varied among children with BM. Sodium level was the main deciding factor for instituting hyponatremia treatment. Most hyponatremia cases responded well to treatment. We found that a negative sodium response at 24 h after treatment initiation and minimum GCS score ≤ 8 predicted dysnatremia at 48 h after treatment initiation. We also found that persistent moderate or severe hyponatremia or hypernatremia at 48 h after the initiation of sodium-altering therapies was uncommon, but often suggested increased disease severity and adverse clinical outcomes in pediatric BM. This highlights the importance of early identification and the institution of appropriate treatment for moderate or severe hyponatremia or hypernatremia in children with BM. Furthermore, the association between hyponatremia correction and short-term adverse clinical outcomes emphasizes the need for future prospective trials to explore a possible causal relationship.

## Data availability statement

The raw data supporting the conclusions of this article will be made available by the authors, without undue reservation.

## Ethics statement

The studies involving human participants were reviewed and approved by the Research Ethics Boards at the Second Affiliated Hospital of Wenzhou Medical University. Written informed consent from the participants' legal guardian/next of kin was not required to participate in this study in accordance with the national legislation and the institutional requirements.

## Author contributions

Conceptualization: FZ, XY, and ZheL. Methodology and formal analysis: FZ. Data curation: FZ, XY, YC, XS, and ZhoL. Writing—original draft preparation: FZ and XY. Writing—review and editing: FZ and ZheL. All authors contributed to the article and approved the submitted version.

## Funding

This work was supported by the Wenzhou Science and Technology Bureau of Zhejiang Province (Nos. Y20210306 and Y20180259).

## Conflict of interest

The authors declare that the research was conducted in the absence of any commercial or financial relationships that could be construed as a potential conflict of interest.

## Publisher's note

All claims expressed in this article are solely those of the authors and do not necessarily represent those of their affiliated organizations, or those of the publisher, the editors and the reviewers. Any product that may be evaluated in this article, or claim that may be made by its manufacturer, is not guaranteed or endorsed by the publisher.
